# Is convalescent plasma futile in COVID-19? A Bayesian re-analysis of the RECOVERY randomized controlled trial

**DOI:** 10.1016/j.ijid.2021.06.034

**Published:** 2021-08

**Authors:** F.W. Hamilton, Todd Lee, D.T. Arnold, R. Lilford, K. Hemming

**Affiliations:** a MRC-IEU Integrative Epidemiology Unit, University of Bristol, Bristol, UK; b Infection Science, North Bristol NHS Trust, Bristol, UK; c Clinical Practice Assessment Unit, Department of Medicine, McGill University, Montreal, Canada; d Academic Respiratory Unit, North Bristol NHS Trust, Bristol, UK; e Institute of Applied Health Research, University of Birmingham, Birmingham, UK

**Keywords:** serology, convalescent plasma, antibody, COVID-19

## Abstract

**Background:**

Randomized trials are generally performed from a frequentist perspective, which can conflate absence of evidence with evidence of absence. The RECOVERY trial evaluated convalescent plasma for patients hospitalized with coronavirus disease 2019 (COVID-19) and concluded that there was no evidence of an effect. Re-analysis from a Bayesian perspective is warranted.

**Methods:**

Outcome data were extracted from the RECOVERY trial by serostatus and time of presentation. A Bayesian re-analysis with a wide variety of priors (vague, optimistic, sceptical, and pessimistic) was performed, calculating the posterior probability for: any benefit, an absolute risk difference of 0.5% (small benefit, number needed to treat 200), and an absolute risk difference of one percentage point (modest benefit, number needed to treat 100).

**Results:**

Across all patients, when analysed with a vague prior, the likelihood of any benefit or a modest benefit with convalescent plasma was estimated to be 64% and 18%, respectively. The estimated chance of any benefit was 95% if presenting within 7 days of symptoms, or 17% if presenting after this. In patients without a detectable antibody response at presentation, the chance of any benefit was 85%. However, it was only 20% in patients with a detectable antibody response at presentation.

**Conclusions:**

Bayesian re-analysis suggests that convalescent plasma reduces mortality by at least one percentage point among the 39% of patients who present within 7 days of symptoms, and that there is a 67% chance of the same mortality reduction in the 38% who are seronegative at the time of presentation. This is in contrast to the results in people who already have antibodies when they present. This biologically plausible finding bears witness to the advantage of Bayesian analyses over misuse of hypothesis tests to inform decisions.

## Highlights

• The RECOVERY trial reported that convalescent plasma had no effect on mortality in COVID-19.
• A re-analysis of the data using Bayesian methods suggests there is a real possibility of benefit.
• The probability of any benefit of plasma in seronegative patients in this trial was more than 75%.

## 1 Introduction

Convalescent plasma (CP) – blood components from patients who have recovered from an infection – has been used for more than a century to treat infections, with widespread use in the 1920s and 1930s for pneumococcal infections and scarlet fever, before falling out of favour with the development of antibiotics (The Lancet Haematology, 2020). The principle is that of ‘passive immunization’, i.e., passing antibodies from those who have recovered from the infection to those naïve to it, thereby providing a degree of protection from that specific agent (Keller and Stiehm, 2000). It is therefore unsurprising that interest in the use of CP to prevent and treat coronavirus disease 2019 (COVID-19) has been widespread (The Lancet Haematology, 2020). Unfortunately, despite best efforts, most of this usage has occurred outside of randomized controlled trials (RCT), with >100 000 doses given in the United States alone (FDA, 2021).

Fortunately, the RECOVERY collaborative group have recently reported the largest RCT of CP in hospitalized patients with COVID-19 (The RECOVERY Collaborative Group et al., 2021). The authors concluded that CP provided no benefit, with the observed mortality equal in both arms: 1399 (24%) of 5795 patients allocated to CP and 1408 (24%) of 5763 patients allocated to usual care died within 28 days (rate ratio (RR) 1.00, 95% confidence interval (CI) 0.93–1.07; *P* = 0.95). They also concluded that there was no difference across pre-specified subgroups, including those with detectable severe acute respiratory syndrome coronavirus 2 (SARS-CoV-2) antibody tests at the time of randomization (seropositive group) (19% versus 18%; RR 1.06, 95% CI 0.94–1.19) and seronegative patients (32% versus 34%; RR 0.96, 95% CI 0.85–1.07), with test for interaction *P* = 0.23. In particular, they noted, on the advice of the Drug Safety and Monitoring Committee (DMC), that: “there was no convincing evidence that further recruitment would provide conclusive proof of worthwhile mortality benefit either overall or in any pre-specified subgroup”. In the United Kingdom, the data have been taken by the regulator as strong evidence of a null effect, leading the Medicines Health Regulatory Authority (MHRA (CAS-ViewAlert 2021); the UK medicines regulator) to recommend against the use of CP in patients hospitalized with COVID-19, effectively removing the therapy in the National Health Service (NHS), with many editorials agreeing with the authors that this proves no effect (Liu and Aberg, 2021).

Before accepting that CP is ineffective in hospitalized patients, it is important to recognize the clear distinction between patients who are likely to benefit and those who are not. The therapeutic mechanism of CP and monoclonal antibody (e.g., REGN-COV2) treatments is passive immunization – the gifting of antibodies. These antibodies (donated by recovered patients) develop in most people by 7–10 days, as part of the normal immune response. It is not surprising to think that the greatest (or any) benefit of CP would only occur in patients who present early or are seronegative, or conversely, that there will be little to no benefit in giving antibodies to those who already have antibodies or have developed their own immune response. The previous literature on severe acute respiratory syndrome (SARS) supports this distinction (Cheng et al., 2005; Yeh et al., 2005), as well as data clearly identifying a protective effect of monoclonal antibodies (manufactured antibodies, rather than donated) in early COVID-19 trials, with much weaker effects in hospitalized patients later in the disease course (ACTIV-3/TICO LY-CoV555 Study Group et al., 2021; Chen et al., 2021; O'Hare, 2021; Weinreich et al., 2021). Immunological data and cases of persistent infection show that failure of an early antibody response is associated with both severe disease and, in patients without any antibodies, the risk of persistent disease (Kemp et al., 2021; Sette and Crotty 2021). Others have also argued that seropositivity is a reason for failure of CP (Bajpai et al., 2020).

On that background, it is logical to analyse the data from patients who are seronegative (hypothesized more likely to benefit) separately from those who are seropositive (hypothesized less likely to benefit). Likewise, it is rational to analyse the data by time from symptom onset, given that the only positive trial of CP occurred with treatment given within 72 hours (Libster et al., 2021).

Although subgroup analyses can be complicated by chance imbalances, lower power, and issues of multiple testing, they are appropriate to generate hypotheses and could be used in support of the argument of not disregarding CP as a potential treatment too soon (Jones et al., 2011; Lee et al., 2021). Moreover, conflating absence of evidence for a small effect with evidence of no effect further risks discarding a therapy that could still have a meaningful benefit. It was therefore sought to undertake a Bayesian re-analysis to estimate the probability of (a) any benefit, (b) a small benefit (defined here as equivalent to a number needed to treat (NNT) of at most 200), and (c) a modest benefit (equivalent of a NNT of at most 100) for all patients and for both subgroups specified above.

## 2 Methods

The intention-to-treat results from the RECOVERY trial were extracted, both overall and for pre-specified subgroups: seronegative, seropositive, ≤7 days since symptom onset, and >7 days since symptom onset. These two subgroups (antibody status and time from onset) were selected on the basis of the scientific justification described above. No granular data were available to combine these two subcategories.

The ‘Bayes’ function Stata version 16 (StataCorp, College Station, TX, USA) was used to calculate posterior probabilities. The probabilities of (a) any benefit (Odds Ratio, OR <1), (b) a small but arguably clinically important benefit, estimated as an absolute risk difference of at least 0.5% (Number Needed to Treat,NNT ≤200), and (c) a modest benefit, which was defined here as a risk difference of at least 1% (NNT ≤100) were calculated. These risk differences were chosen after internal discussion between the study authors regarding what would be considered an important effect size considering the complexity and challenges in using CP. By nature, they are subjective, but reflect effect sizes that might be salient to patients, their families, and clinicians.

As suggested by a recent review on Bayesian re-analysis in COVID-19 (Zampieri et al., 2021), four probability assumptions were chosen to account for varying prior views: (1) vague (no information; mean risk difference (RD) 0, standard deviation (SD) 10 000); (2) optimistic (10% risk of harm; mean RD 0.01, SD 0.007); (3) sceptical (tightly around the null; mean RD 0, SD 0.007); (4) pessimistic (10% chance of benefit; mean RD –0.005, SD 0.0036). Posterior probabilities were computed from binomial regression models. Posterior density function graphs were produced for each prior assumption.

All code used to generate these figures is available in the **Supplementary Material**.

## 3 Results

Table 1 presents the posterior probabilities of benefit for each prior.

**Table 1:** Estimated posterior probabilities of benefit for a variety of prior assumptions

	Vague prior	Optimistic prior	Sceptical prior	Pessimistic prior
Whole trial (*n* = 11 558)				
Any benefit	64%	65%	64%	62%
Small benefit	43%	41%	40%	38%
Modest benefit	20%	19%	19%	18%
Seronegative subgroup (*n* = 3676)				
Any benefit	86%	87%	86%	86%
Small benefit	79%	78%	77%	78%
Modest benefit	68%	68%	66%	67%
Seropositive subgroup (*n* = 5888)				
Any benefit	20%	23%	21%	21%
Small benefit	9%	11%	11%	10%
Modest benefit	3%	4%	4%	4%
≤7 days since symptom onset (*n* = 4466)				
Any benefit	95%	95%	95%	95%
Small benefit	90%	90%	90%	90%
Modest benefit	80%	82%	82%	80%
>7 days since symptom onset (*n* = 7086)				
Any benefit	17%	17%	17%	17%
Small benefit	7%	8%	7%	7%
Modest benefit	3%	3%	2%	2%

Vague prior: N(0, SD = 10 000); optimistic prior: N(0.01, SD = 0.007); sceptical prior: N(0, SD = 0.007); pessimistic prior N(−0.01, SD = 0.0036). Small benefit defined as a risk difference >0.5% (equivalent to a NNT ≤200); Moderate benefit defined as a risk difference >1% (equivalent to a NNT ≤100). SD, standard deviation; NNT, number needed to treat.

Across the whole trial population, the estimated chance of any benefit was found to be around 65%, with little difference across all prior assumptions. The posterior probability of a modest benefit (preferring treatment arm) was found to be around 19% across all prior assumptions. The associated posterior density functions are available in the **Supplementary Material**, as supplementary figures.

In the seronegative subgroup, the estimated likelihood of any benefit was greater, at around 90%, across all prior assumptions. The estimated chance of a risk difference (modest benefit) of >1% was also high (more than 66% across all three priors), and varied little between prior assumptions. This contrasts with the seropositive arm, where the estimated chance of any benefit was only 20%, and with a very small (3%) chance of a modest benefit (NNT ≤100).

These results are mirrored in the early treatment subgroup, with an around 95% chance of benefit in patients treated within 7 days of symptom onset. The chance of a modest benefit (NNT ≤100) was about 80% across all prior assumptions. However, in patients who presented after 7 days, the chance of CP providing any benefit was small (17%), with a very low chance (~2%) of a modest benefit (NNT ≤100).

## 4 Discussion

The RECOVERY trial has been a paradigm for a rapid pragmatic approach to trialling new therapies in a pandemic. Good practice requires a firm pre-specified analysis plan with a clear pre-defined subgroup analysis (Schulz et al., 2010). However, the conclusions drawn by the RECOVERY collaborative group and the MHRA with respect to CP risks conflating absence of evidence of a small effect with evidence that there is no benefit. Re-analysis of the original data using Bayesian methods yielded a small probability (>15%) of an effect with an NNT of 100 across the whole trial, with even higher probabilities of 90% and 75%, respectively, in patients presenting within 7 days of symptoms and patients antibody-negative on presentation.

Patients in the population who presented early were easy to identify (from history alone) and constituted more than a third of the whole trial population. The estimated chance of a benefit with a NNT of 100 changed from ~7% in those presenting late to ~90% in those presenting early.

Many clinicians, patients, and their families might consider benefits in the region of one life saved for every 100 or 200 people treated as meaningful benefits. From a societal perspective, the treatment would need to achieve a mean of only one quality-adjusted life year to justify a £20 000 treatment cost. However, it is not our intention to prove that CP is a cost-effective treatment – at heart that is a value judgement. We wish only to show that the conclusion that the treatment is ineffective is unlikely to be true for people who have not developed immunity at the point where the therapeutic decision is made. It is always important to consider the literature in the round when making policy recommendations.

Previous trials have been small and underpowered, with a recent meta-analysis of evaluations of CP in COVID-19 identifying less than 2000 patients across all RCTs prior to RECOVERY (Janiaud et al., 2021). Only one previous trial of high-titre CP reported data based on antibody status (Simonovich et al., 2021), with 34/105 deaths (Rodionov et al., 2021) in the seronegative placebo arm and 65/228 deaths in the CP arm, a RR of death with CP of 1.12, but with very wide confidence intervals (95% CI 0.51–2.43). A further trial, which stopped early due to declining case numbers, recruited older adults within 72 hours of symptoms (Libster et al., 2021). In that study, 13/80 patients (16%) who received CP developed severe disease compared to 25/80 (31%) in the placebo arm, giving a RR of 0.52 (95% CI 0.29–0.94) in favour of CP. In a recent case series of 14 immunocompromised patients with COVID-19 who had no detectable SARS-CoV-2 IgG, transfusion of CP was associated with clinical improvement and the degree of clinical improvement correlated with the IgG titre post transfusion. Finally, the largest observational study in hospitalized patients (*n* = 3082), a US registry study (published after RECOVERY), identified a lower risk of death in patients transfused early with higher levels of SARS-CoV-2 IgG antibody. Taken in the round, the literature supports the present re-analysis of the RECOVERY data, showing a benefit of CP among immunologically naïve patients with COVID-19 (Joyner et al., 2021).

Yet further support for our conclusion can be found from secondary outcomes in the RECOVERY trial that we would expect to correlate with the primary outcome if the hypothesis that CP is particularly effective in immunologically naïve patients. Both secondary outcomes in the original study, i.e., discharge home by day 28 and invasive mechanical ventilation or death, showed heterogeneity with respect to serological status and intervention effect, with impressive *P*-values of 0.008 and 0.01, respectively, in favour of CP. Although we have not focused on this to avoid accusations of ‘cherry picking’ the data, this is entirely consistent with and supportive of a causal path by which CP reduces mortality, and both of these are critical outcomes relevant to both patients and clinicians.

It is recognized that there may have been chance imbalances in age or comorbidity within the seronegative or early subgroup of patients, since randomization was not stratified on serological status or time from presentation (The RECOVERY Collaborative Group et al., 2021). However, they were pre-specified subgroups and made up a substantial proportion of all participants as stated above. It is also recognized that both of these groups double counted a number of patients for the reason given above. An alternative approach would have been to create four non-overlapping groups: both (serologically positive and late presentation), neither, and two either groups. However, these groups could not be constructed because we did not have the raw data. In any event, both serological negativity and early presentation tap into a shared pathway concerning the development of immunity, even if there are other pathways involved.

In conclusion, the RECOVERY trial for CP reported no benefit. Recognizing the changing literature since the trial started and using a variety of priors, we suggest the reporting of no effect may be premature. It remains plausible that CP has a small but clinically important effect on mortality in those who have not already developed an antibody response or who present early. It is clear that any effect is likely small, but we would argue that clinicians, scientists, and government agencies should review all trial data in totality, rather than regarding the null result as fixed.

## Appendix Supplementary materials

Image, application 1
