# Supplementary material for: Is convalescent plasma futile in COVID-19? A Bayesian re-analysis of the RECOVERY randomized controlled trial
Source: Int J Infect Dis. 2021 Aug;109:114–7. doi: 10.1016/j.ijid.2021.06.034 (PMC8214317; doi:10.1016/j.ijid.2021.06.034)

Supplementary Appendix 1:

Figure S1: Posterior probability density function for all patients


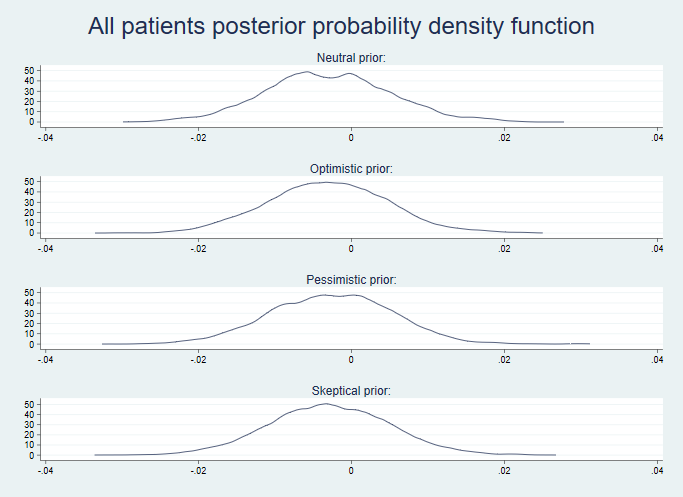


Figure S2: Posterior probability density function for seronegative patients


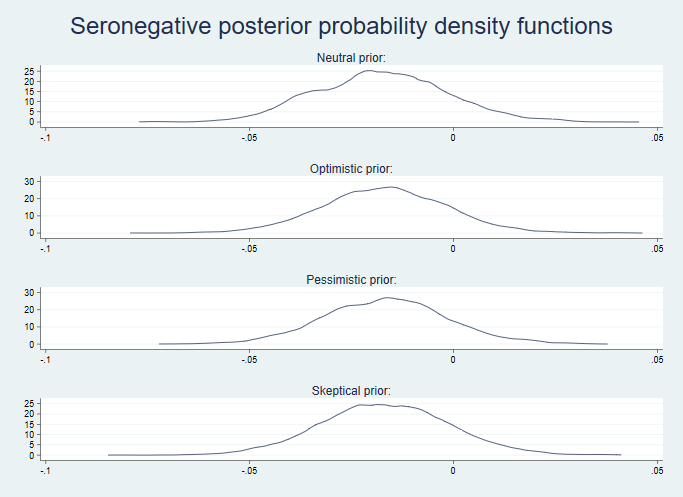


Figure S3: Posterior probability density function for seropositive patients


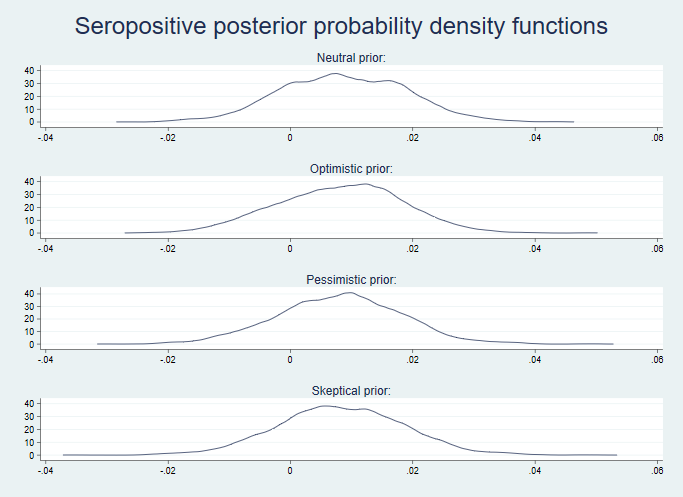


Figure S4: Posterior probability density function for patients admitted within 7 days


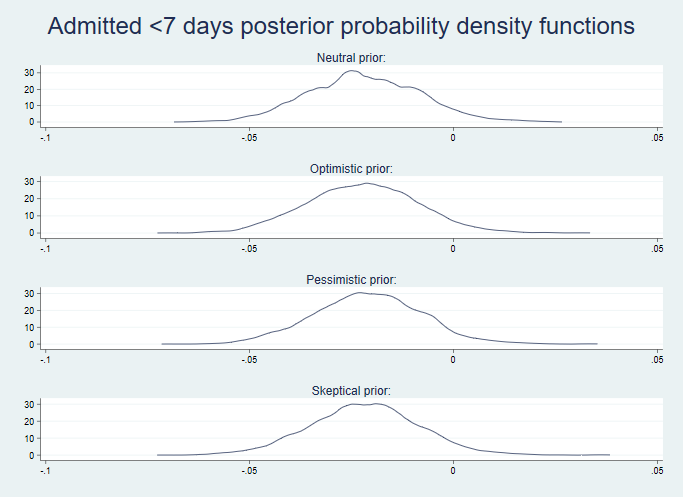


Figure S5: Posterior probability density function for patients admitted after 7 days


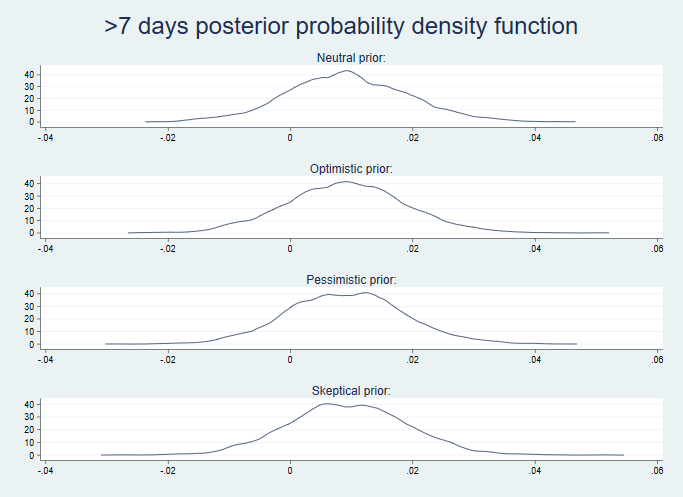

Supplement: Supplementary file 1 [file mmc1.docx]
